# Enhancement of surface tribology, mechanical, and electrical properties of UHMWPE *via* graphene nanoplatelets coating and electron beam irradiation

**DOI:** 10.1039/d5ra04349b

**Published:** 2025-07-28

**Authors:** Thitisorn Anekratmontre, Doonyapong Wongsawaeng, Grittima Kongprawes, Donruedee Toyen

**Affiliations:** a Department of Nuclear Engineering, Faculty of Engineering, Chulalongkorn University 254 Phayathai Road, Pathumwan Bangkok 10330 Thailand Doonyapong.W@Chula.ac.th; b Thailand Institute of Nuclear Technology (Public Organization) 9/9 Moo 7, Sai Mun Ongkharak Nakhon Nayok 26120 Thailand; c Department of Materials Science, Faculty of Science, Kasetsart University Bangkok 10900 Thailand

## Abstract

This work aims to investigate the enhancement of tribological, mechanical, and electrical properties of ultra-high molecular weight polyethylene (UHMWPE) through surface modification *via* graphene nanoplatelet (GNP) coating combined with electron beam (E-beam) irradiation. UHMWPE substrates were dip-coated with 1 wt% GNPs and subjected to E-beam irradiation at doses ranging from 0 to 500 kGy. Among the tested conditions, irradiation at 100 kGy yielded the most favorable outcomes, including a reduced coefficient of friction (0.1793), improved tensile strength (28.94 MPa), increased elongation at break (58.35%), and the highest surface hardness (68 Shore D). Furthermore, the surface resistivity decreased markedly to 2.15 × 10^8^ Ω, indicating a significant improvement in surface conductivity. Fourier-transform infrared spectroscopy (FTIR) revealed the formation of carbonyl groups (C=O), attributed to oxidative processes initiated by irradiation-induced free radicals. Scanning electron microscopy (SEM) images confirm enhanced GNP adhesion and uniform dispersion at moderate irradiation levels. However, excessive irradiation doses, exceeding 100 kGy, led to the degradation of both structural and functional properties due to polymer chain scission. These findings demonstrate that the synergistic integration of graphene coating and optimally tuned E-beam irradiation, particularly at 100 kGy, offers a promising strategy for developing UHMWPE-based materials with superior multifunctional performance for advanced tribological, mechanical, and electrical applications.

## Abstract 2

This work aims to study the enhancement of tribological, mechanical, and electrical properties of ultra-high molecular weight polyethylene (UHMWPE) *via* surface modification using graphene nanoplatelet (GNP) coating and electron beam irradiation.

## Graphical abstract

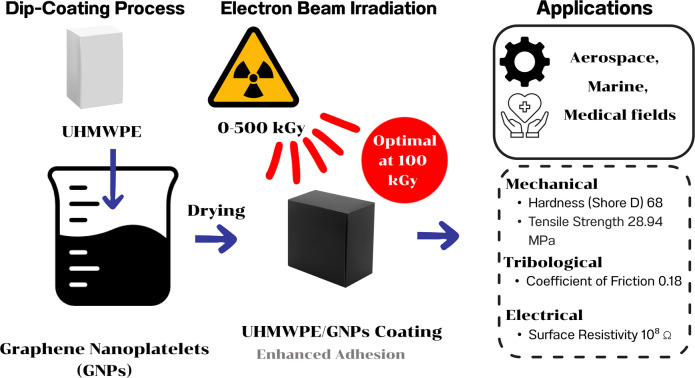

## 1 Introduction

The development and improvement of polymer materials for various applications, especially in electronics, biomedicine, and in the radiation protection industry, require the adjustment of specific properties of the materials. One of the materials that has received much attention is Ultra-High Molecular Weight Polyethylene (UHMWPE). Typically ranges from 3–6 million g per mol or more. This type of polymer is a plastic that has many outstanding properties. It has high wear resistance, high mechanical strength, resistance to chemical corrosion, and a low coefficient of friction. Therefore, it is widely used in industry.^1^ For example, in medical applications, UHMWPE is used in artificial knee joints (orthopedic implants), and in automotive engineering, UHMWPE is used to make gears, bearings, rollers, and force support plates. In the sports industry, UHMWPE is used in snowboard slides or ice hockey. However, the problem of surface deterioration remains to be considered, mainly when used in an environment where there are forces or external factors that affect the molecular structure of the material, such as oxidative degradation, UV degradation, wear and abrasion degradation.^2^

We can effectively solve this issue with techniques such as molecular crosslinking using electron beam radiation, which enhances structural strength and reduces wear. The electron beam is a high-energy radiation that ionizes certain bonds in the polymer chain, causing the molecules to form a crosslink.^3^ In addition, developing materials with increased strength and wear resistance can utilize surface modification methods. For example, surface coating or improvement using graphene coating^4^ and diamond-like carbon (DLC) coating. Adding or coating with graphene enhances mechanical strength and wear resistance and significantly reduces friction. Graphene is an extreme material with self-lubricating properties. It is ideal for applications that require resistance to pressure and long-term scratching, such as medical device components or equipment that rotates or moves continuously.^5^ DLC coating with a carbon layer yields a surface structure similar to diamond, significantly reducing friction, resulting in a smoother surface, decreased wear, and extended lifespan. This is ideal for components that experience continuous movement or rotation, such as ball bearings or prosthetic joints.^6^ In addition, one of the most convenient methods for applying a coating to a surface is dip coating, which is a process in which a workpiece material is dipped into a solution or suspension containing a coating material, drawn upwards at a controlled rate, and allowed to dry or bake to adhere the coating material to the surface of the workpiece.^7^ The basic steps of this process consist of three main steps: immersion, withdrawal, and drying (or curing)—control of withdrawal speed, coating viscosity, such as glass fibers or various polymer materials. The number of dipping cycles affects the thickness and uniformity of the coating. These include process simplicity, the ability to control the thickness of the coating by adjusting the number of dipping cycles, and the ability to coat complex surface shapes,^8^ such as glass fibers or various polymer materials.

Graphene nanoplatelets (GNPs) are carbon nanomaterials that have garnered significant attention due to their unique properties, including high electrical and thermal conductivity, excellent mechanical strength,^9^ and a high area-to-volume ratio. This makes GNPs potentially valuable for many industries requiring high-barrier, electrical conductivity, and durability properties. GNPs are multi-layered sheets of graphene, typically 1–10 nm thick, and range in diameter from 100 nm to several micrometers.^10^ The structural characteristics of GNPs are attributed to the presence of carbon atoms interconnected by sp^2^ covalent bonds, which results in high strength and good chemical stability.^11^ GNPs can be utilized in surface coating applications by incorporating them into polymers, coating agents, or coating solutions, decreasing wear rate^4^ and enhancing their resistance to chemicals and harsh environments. They can be applied to a wide range of materials.

Pristine UHMWPE exhibits strong electrical insulation properties, limiting its application in environments requiring conductive properties. Coating UHMWPE with GNPs has become an effective method for enhancing its properties. Since graphene is an excellent conductive material with a two-dimensional crystal structure that allows electrons to move freely, the graphene coating can change the behavior of the UHMWPE surface from an insulator to a conductive material to some extent.^12^ However, graphene coating cannot be chemically bonded together because UHMWPE has low adhesion. Electron Beam Irradiation (EBI) is a popular process for modifying polymer structures, such as crosslinking or surface modification.^3^ It has the advantages of increasing strength, thermal stability, and wear resistance. It was confirmed by previous studied, for example, Zhang *et al.*^13^ developed a SiO_2_–graphene oxide reinforced epoxy coating cured by electron beam irradiation. This significantly improved corrosion resistance by enhancing the coating's crosslinking density and barrier properties. Ma *et al.*^14^ investigated the effects of low-energy electron beam irradiation on colloidal siloxane-coated polymer films. Using this technique, they promoted effective polymerization and crosslinking within the siloxane coating. As a result, the surface hardness of the films increased significantly. Their study demonstrated that electron beam curing is a highly efficient method for enhancing the mechanical performance and surface durability of polymer coatings.

EBI technology is a significant innovation in various industries. It utilizes high-energy electron beams to perform physical and chemical processes, including welding, cutting, surface treatment, and writing patterns on the nanometer scale.^15^ This technology is key in the semiconductor, metal fabrication, and medical industries. The E-beam system emits high-energy electrons from an electron gun, typically consisting of a tungsten filament heated to produce electron emissions. Therefore, this work aims to advance further the progress in developing UHMWPE products *via* graphene coating on the surface using the EBI technique. This assists in enhancing GNP adhesion and inducing beneficial changes in the polymer structure, offering a promising approach for advanced material development, but also improves the surface, mechanical, and electrical properties of UHMWPE.

## 2 Experimental sections

### 2.1 Preparation of UHMWPE/graphene coating samples and E-beam irradiation

UHMWPE was acquired from the Ottensteiner Kunststoff GmbH & Co. KG, Germany, with dimensions of 16.5 cm × 16.5 cm and a thickness of 3 mm. After that, the surface of UHMWPE was coated. The technique to coat this material is dip coating, following the method described by Pietrzyk, B. *et al.*^16^ The process involved preparing a 1 wt% dispersion of GNPs in 100 mL of ethanol using an ultrasonic machine operated at a power of 180 W and a frequency of 40 kHz for 30 min at 30 °C. Ultrasonication effectively promoted the homogeneous dispersion of GNPs in ethanol, as supported by previous studies, making the suspension suitable for uniform dip-coating. The UHMWPE sheets were then immersed in the GNP solution for 5 min and baked at 80 °C for 24 h. After that, the UHMWPE/graphene coating samples were E-beam irradiated at Thailand Institute of Nuclear Technology (TINT), Thailand, with a varying dose at 0, 50, 100, 150, 200, and 250 kGy.

### 2.2 Tribological properties

Wear resistance was evaluated by measuring the coefficient of friction (coefficients of friction) of pin on disk using Universal Tribotester (Ducom, USA) type referring to ASTM G99-95a^17^ in dry condition with a high-chromium steel ball diameter of 6.35 mm, a pressing weight of 5 N, a speed of 0.3 m s^−1^, a sliding distance of 1000 m, the test diameter is equal to 10 mm. The samples were cut into a square of 43 mm × 43 mm and 3 mm thick.

### 2.3 Morphology and elemental composition analysis

The determination of morphology, the dispersion of GNPs, the adhesion of GNPs on the surface of UHMWPE, and the elemental composition of the UHMWPE/GNPs coating was conducted using scanning electron microscopy (SEM) (FEI; Quanta 450, Czechia). To determine the morphologies of the adhesion surface, all samples were coated with platinum for 30 seconds to improve electrical conductivity on the sample surface and avoid effects of charge accumulation that may distort or reduce image quality.

### 2.4 Mechanical properties

Mechanical properties of sample, consisting of the tensile modulus, tensile strength, and elongation at break, were assessed using a Universal Testing Machine (TM-G5K; TM Tech Testing Co., Ltd; Bangkok, Thailand).^18^ Tensile testing was performed at a speed of 100 mm min^−1^. The samples were tested using a durometer (GS-719G; Teclock; Nagano, Japan) according to ASTM D2240-03 (Shore D)^19^ standard testing for the surface hardness measurements. Notably, at least 3 repetitions for each formulation were carried out for all mechanical measurements.

### 2.5 Electrical properties

The surface resistivity can be calculated using eqn (1). The DC voltage released from the high-voltage supply passes through the concentric ring electrode, then through the sample surface, into the concentric circle electrode, and the DC voltage will pass into the electrometer to obtain the current value according to ASTM D257.^20^ The samples were cut into a circle with a diameter of 3.5 inches and a thickness of 2 mm.1
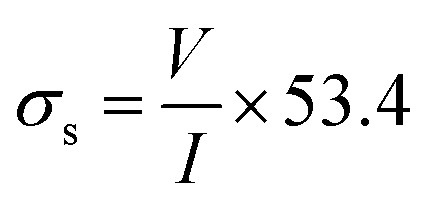
where *σ*_s_ is surface resistivity (Ω), *V* is voltage (V), *I* is current (A).

### 2.6 Contact angle

The contact angle was measured using a contact angle goniometer (Kino; PUDITEC Co., Ltd; Bangkok, Thailand) type, referring to ASTM D7334-08.^21^ The contact angle measurement process begins with sample preparation. The samples were cut into a square with a diameter of 1 cm and a thickness of 3 mm. The surface should be clean and free from contamination, dust, or grease. Then, a small amount of water, approximately 2 to 5 microliters, is carefully dropped onto the surface. When a water drop falls on a surface, an image of the drop is immediately taken from the side to clearly show the shape of the drop and the contact line between the liquid and the solid. The resulting image is then analyzed.

### 2.7 Fourier transform infrared spectroscopy

Functional groups of UHMWPE, UHMWPE/graphene coating with E-beam irradiation at 0–500 kGy doses were analyzed using Fourier transform infrared spectroscopy (FTIR) with Universal Attenuated Total Reflectance (UATR) sensor technique the resolution, scan range, and number of sample scans were 4.0 cm^−1^, 4000–515 cm^−1^, and 8, respectively.

## 3 Results and discussion

### 3.1 Molecular structure

**Fig. 1:**
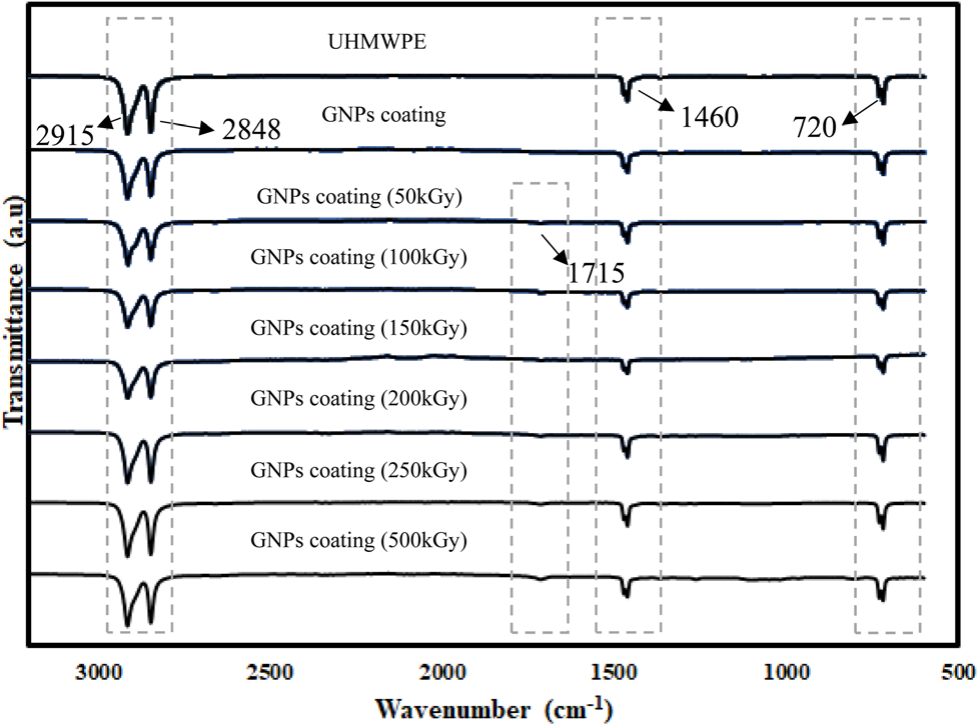
FTIR spectra of UHMWPE, UHMWPE/graphene coating with E-beam irradiation at 0, 50, 100, 150, 200, 250, 500 kGy, respectively.

**Fig. 2:**
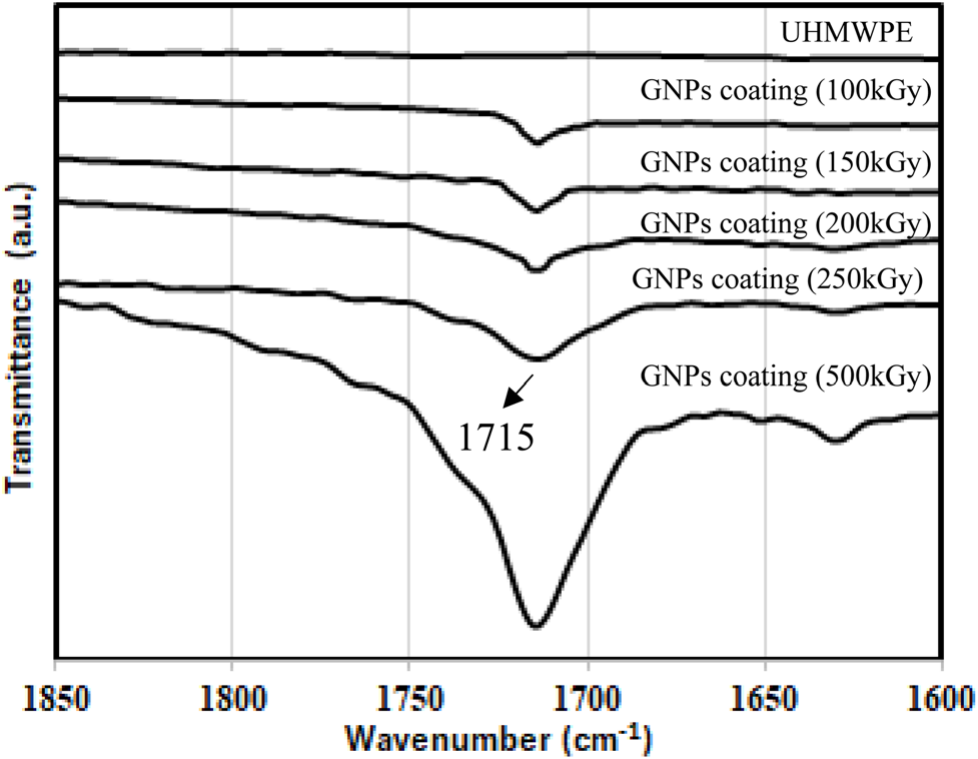
FTIR spectra of UHMWPE, UHMWPE/graphene coating with E-beam irradiation at 100, 150, 200, 250, 500 kGy, respectively.

The FTIR spectra of the UHMWPE, UHMWPE/graphene coating with E-beam irradiation at 0, 50, 100, 150, 200, 250, and 500 kGy are shown in Fig. 1. For the spectra of UHMWPE and UHMWPE/graphene coating with E-beam irradiation 0 kGy, the peaks were observed at 720–730, 1460–1470, 2848, and 2915 cm^−1^, corresponding to CH_2_, C–H, and C–H, respectively. This is because UHMWPE/graphene coating with E-beam at 0 kGy did not form chemical bonds. The FTIR spectra of UHMWPE/graphene coatings subjected to electron beam irradiation from 0 to 500 kGy exhibit a distinct but initially weak absorption band at approximately 1715 cm^−1^, corresponding to the C=O stretching vibration. This feature becomes increasingly prominent with higher doses, indicating that E-beam irradiation leads to bond cleavage within the UHMWPE matrix, particularly of C–C and C–H bonds. The resulting free radicals readily react with atmospheric oxygen, forming carbonyl groups on the polymer surface.^22^ To further quantify this effect, the carbonyl peak was analyzed across the irradiation doses. As shown in Fig. 1, the C=O peak is absent in the unirradiated and 50 kGy samples, begins to appear at 100 kGy, and intensifies progressively at higher doses. This trend clearly demonstrates that oxidation increases in a dose-dependent manner, driven by radiation-induced radical formation and subsequent oxidative reactions.^23^ To reinforce these qualitative observations, a quantitative analysis of the C=O peak was carried out by integrating the area under the absorption band centered at 1715 cm^−1^ (within the 1705–1725 cm^−1^ range) using the Opus software. The integrated areas were then converted into relative percentages, using the UHMWPE control sample as the baseline (0%). The results showed a progressive increase in carbonyl peak intensity by 0.97%, 2.65%, 4.07%, 5.95%, and 7.67% for samples irradiated at 100, 150, 200, 250, and 500 kGy, respectively. This relative increase clearly reflects the growing carbonyl content and confirms the dose-dependent oxidative degradation of the polymer surface. These quantitative results are summarized in Fig. 2 and support the correlation between irradiation dose and oxidation level in the UHMWPE/graphene coatings.

### 3.2 Morphology analysis

**Fig. 3:**
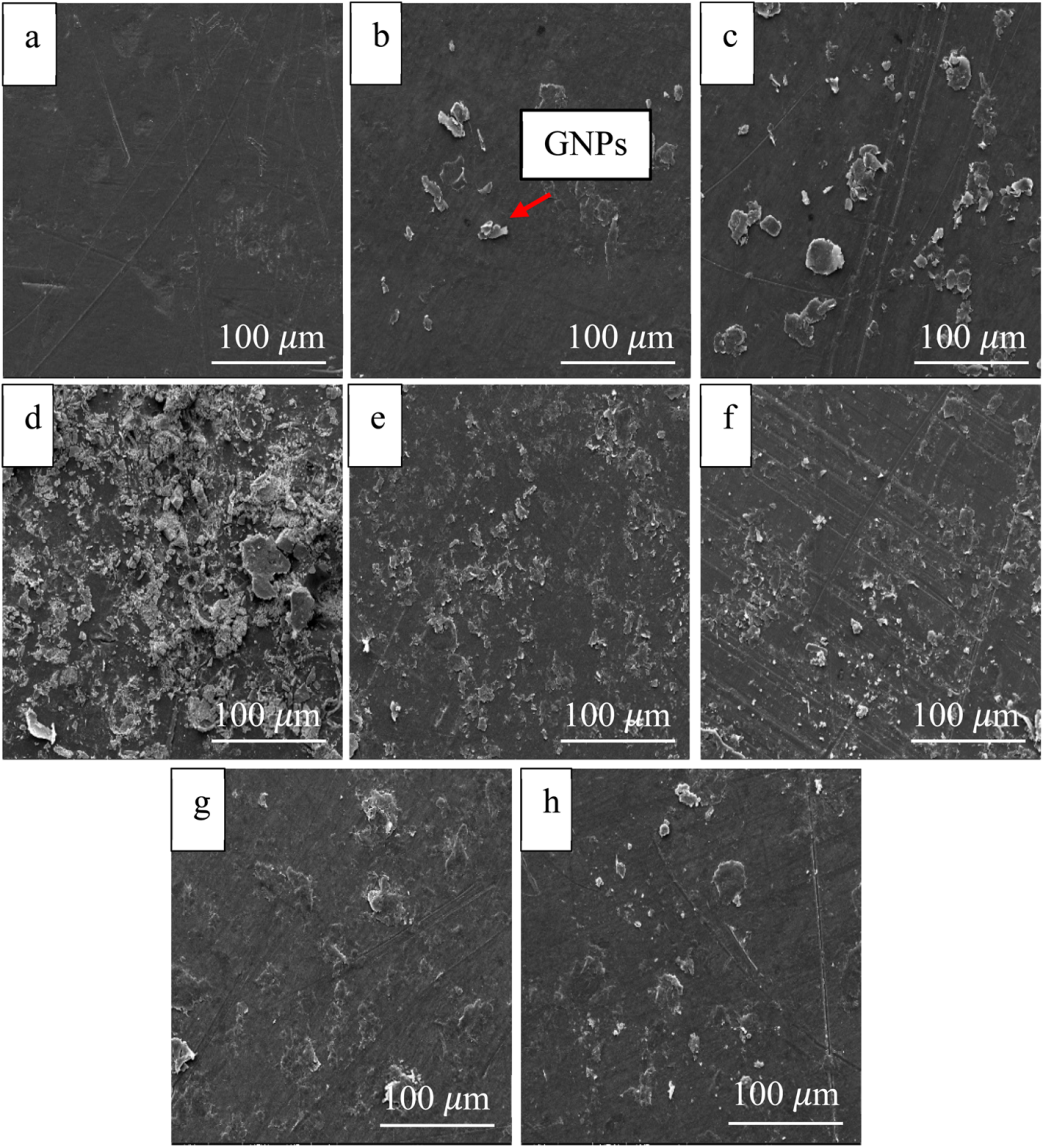
SEM images of (a) UHMWPE, (b–h) UHMWPE/graphene coating with E-beam at 0, 50, 100, 250, and 500 kGy, respectively.

The morphology of the samples was analyzed by SEM, as shown in Fig. 3. The surface structure changes depending on the irradiation dose. The non-irradiated sample (0 kGy) had poor adhesion of GNPs on the surface, resulting in a rough and uneven surface distribution. When the irradiation was increased to 50–100 kGy, the surface adhesion of GNPs was significantly improved due to the formation of chemical bonds between GNPs and the polymers. The dose of 100 kGy provided the best results in terms of both structure and surface properties.^24^ However, at the radiation level beyond 150 kGy, there was the deterioration of the surface structure due to chain scission will start to occur. This caused the GNPs not to adhere tightly and the surface to become rougher, especially at 250–500 kGy, which may adversely affect the strength and stability of the material.^25^

### 3.3 Tribological properties

The results in Table 1 show that the uncoated UHMWPE had a coefficient of friction (CoF) value of 0.29, which was considered low according to the basic properties of the material. The coated sample with GNPs without irradiation, the CoF value significantly increased to 0.62, which could explain the low cohesive strength of the UHMWPE surface. This causes the GNPs not to adhere uniformly, resulting in a rougher surface and increased friction. When the materials were irradiated with E-beam at the dose of 50–500 kGy, the CoF values decreased significantly. The lowest CoF was 0.18 found at a dose of 100 kGy, which is even lower than the baseline value of pristine UHMWPE. This implies that E-beam irradiation promotes crosslinking between UHMWPE molecules. It helps improve the adhesion of GNPs on the surface, resulting in a uniform, smooth surface with excellent friction resistance performance. However, when the dose was increased above 100 kGy, such as 150–500 kGy, the CoF values increased slightly again, although they were still lower than those of unirradiated GNPs. This trend might be because excessive radiation doses cause chain scission or breakdown of polymer chains and coating structures, deteriorating the surface structure and reducing properties from the optimum point.^26^

**Table 1:** Average coefficient of friction of UHMWPE and UHMWPE/GNPs coating, with varying E-beam irradiated contents

Samples (E-beam content kGy)	Average coefficient of friction
UHMWPE uncoating (0 kGy)	0.29 ± 0.002
UHMWPE/GNPs coating (0 kGy)	0.62 ± 0.008
UHMWPE/GNPs coating (50 kGy)	0.40 ± 0.040
UHMWPE/GNPs coating (100 kGy)	0.18 ± 0.007
UHMWPE/GNPs coating (150 kGy)	0.23 ± 0.008
UHMWPE/GNPs coating (200 kGy)	0.22 ± 0.026
UHMWPE/GNPs coating (250 kGy)	0.24 ± 0.027
UHMWPE/GNPs coating (500 kGy)	0.24 ± 0.015

### 3.4 Mechanical properties

The mechanical properties of UHMWPE coated with GNPs and irradiated with varying E-beam doses at 0, 50, 100, 150, 200, 250, and 500 kGy are summarized in Table 2. The results show that the sample irradiated at 100 kGy exhibited the highest overall mechanical performance. This sample had a tensile modulus of 28.67 ± 0.44 MPa, tensile strength of 28.94 ± 0.33 MPa, and elongation at break of 58.35 ± 1.43%, which were significantly higher or comparable to those of the non-irradiated control. In addition, its surface hardness reached the maximum value of 68 Shore D, suggesting improved surface strength.

**Table 2:** Mechanical properties of UHMWPE/GNPs coating, with varying E-beam irradiated contents

E-beam content (kGy)	Tensile modulus (MPa)	Tensile strength (MPa)	Elongation at break (%)	Hardness (Shore D)
0	28.20 ± 0.40	28.31 ± 0.40	59.59 ± 2.08	65 ± 1
50	28.69 ± 0.46	28.77 ± 0.38	47.92 ± 3.51	68 ± 1
100	28.67 ± 0.44	28.94 ± 0.33	58.35 ± 1.43	68 ± 1
150	21.44 ± 0.35	26.31 ± 0.05	25.59 ± 3.58	67 ± 1
200	18.21 ± 1.71	25.44 ± 0.41	39.01 ± 8.92	67 ± 1
250	19.11 ± 1.89	25.16 ± 0.27	23.81 ± 0.07	66 ± 1
500	14.98 ± 0.88	22.28 ± 0.37	48.58 ± 2.38	64 ± 1

These improvements are attributed to the electron beam-induced crosslinking of the UHMWPE chains, which enhances intermolecular bonding, improves load transfer efficiency, and increases resistance to mechanical deformation.^27^ The optimal dose of 100 kGy appears to strike a balance between crosslinking enhancement and avoiding excessive chain scission. However, the mechanical properties declined significantly when the dose was increased above 100 kGy. For example, the 250 kGy and 500 kGy samples exhibited reduced tensile strength and elongation at break, indicating increased molecular chain degradation. This suggests that excessive radiation doses lead to chain scission dominating over crosslinking, which weakens the material structure and reduces its ductility.^28^ Therefore, 100 kGy was identified as the optimal E-beam dose to enhance the mechanical integrity of UHMWPE/GNPs coatings without compromising elasticity and toughness.

**Fig. 4:**
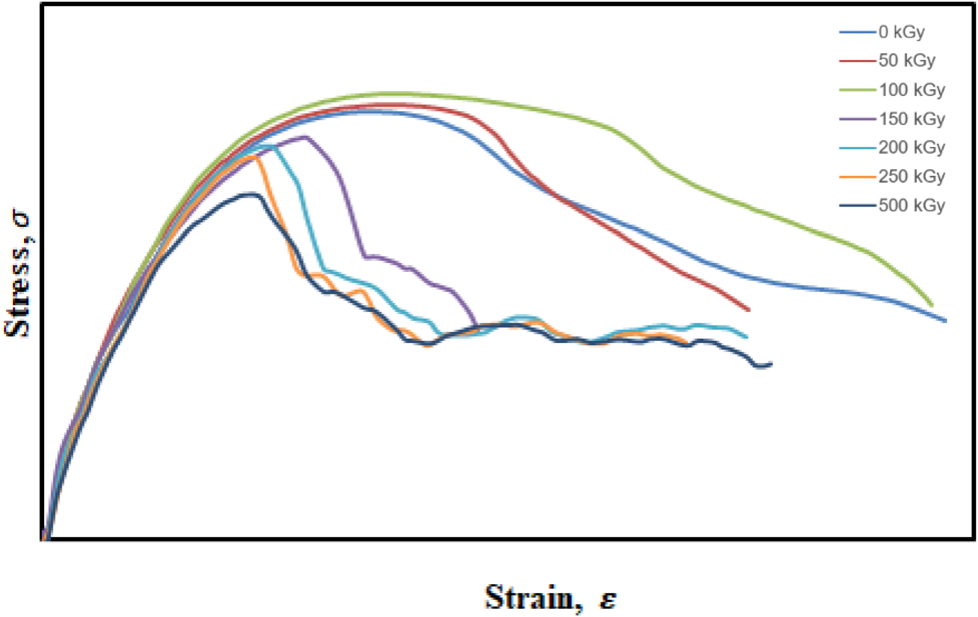
Stress–strain curve of UHMWPE/graphene coating with E-beam at 0, 50, 100, 250, and 500 kGy, respectively.

The stress–strain curves of UHMWPE samples modified with GNPs and subjected to various electron beam irradiation doses demonstrate the evolution of mechanical behavior under tensile loading. All samples exhibit typical characteristics of ductile polymers, including a linear elastic region, yield point, and strain-hardening phase. As shown in Fig. 4, the sample irradiated at 100 kGy exhibited the highest tensile strength and elongation at break, indicating an optimal crosslinking density that enhances both mechanical strength and flexibility. This behavior is attributed to a balance between chain crosslinking and minimal chain scission. In contrast, samples irradiated at higher doses such as 250 and 500 kGy showed reduced mechanical properties, with lower peak stress and decreased strain at break, suggesting degradation due to excessive chain scission. These results are consistent with previous findings on radiation-modified polymers, where mechanical performance improves only within an optimal irradiation window before degradation becomes dominant.^29,30^

### 3.5 Electrical properties

The result of electrical properties is demonstrated in Table 3. It was found that the coating of GNPs on the surface of UHMWPE significantly reduced the surface resistance value, with the resistance value of unirradiated UHMWPE/GNPs reduced to 5.35 × 10^10^ from 5.33 × 10^15^ Ω. This indicated that GNPs play a role in increasing the electrical conductivity on the surface of the material.^31^ However, the adhesion of GNPs to the surface of the material in the non-irradiated state was incomplete, resulting in uneven distribution. The E-beam irradiation at 50 and 100 kGy resulted in the resistance decreased continuously, reaching a minimum value at 100 kGy of 2.15 × 10^8^ Ω. This supported that the E-beam induces crosslinking of UHMWPE molecules. It also promotes better adhesion of GNPs on the material surface, allowing GNPs to arrange themselves into a uniform network, which results in significantly improved electrical conductivity on the material surface. However, when the radiation dose is increased beyond 100 kGy, such as 150–500 kGy, the resistance value increases again, especially at 500 kGy, equal to 1.62 × 1011 Ω, higher than at lower levels. This increase is caused by the high level of radiation, causing chain scission and disruption of GNPs' structure as well as surface damage, which leads to discontinuity of the conducting network and a decrease in the conductive properties.

**Table 3:** Electrical properties, consisting of surface resistivity, of graphene, UHMWPE uncoating, UHMWPE/GNPs coating, with varying E-beam irradiated contents

Samples (E-beam content kGy)	Surface resistivity (Ω)
Graphene (0 kGy)	10^2^–10^5^
UHMWPE uncoating (0 kGy)	(5.22 ± 0.36) × 10^15^
UHMWPE/GNPs coating (0 kGy)	(5.35 ± 0.32) × 10^10^
UHMWPE/GNPs coating (50 kGy)	(2.45 ± 0.24) × 10^8^
UHMWPE/GNPs coating (100 kGy)	(2.15 ± 0.10) × 10^8^
UHMWPE/GNPs coating (150 kGy)	(5.22 ± 0.36) × 10^8^
UHMWPE/GNPs coating (200 kGy)	(1.12 ± 0.26) × 10^9^
UHMWPE/GNPs coating (250 kGy)	(3.59 ± 0.20) × 10^9^
UHMWPE/GNPs coating (500 kGy)	(1.62 ± 0.17) × 10^11^

### 3.6 Contact angle

**Fig. 5:**
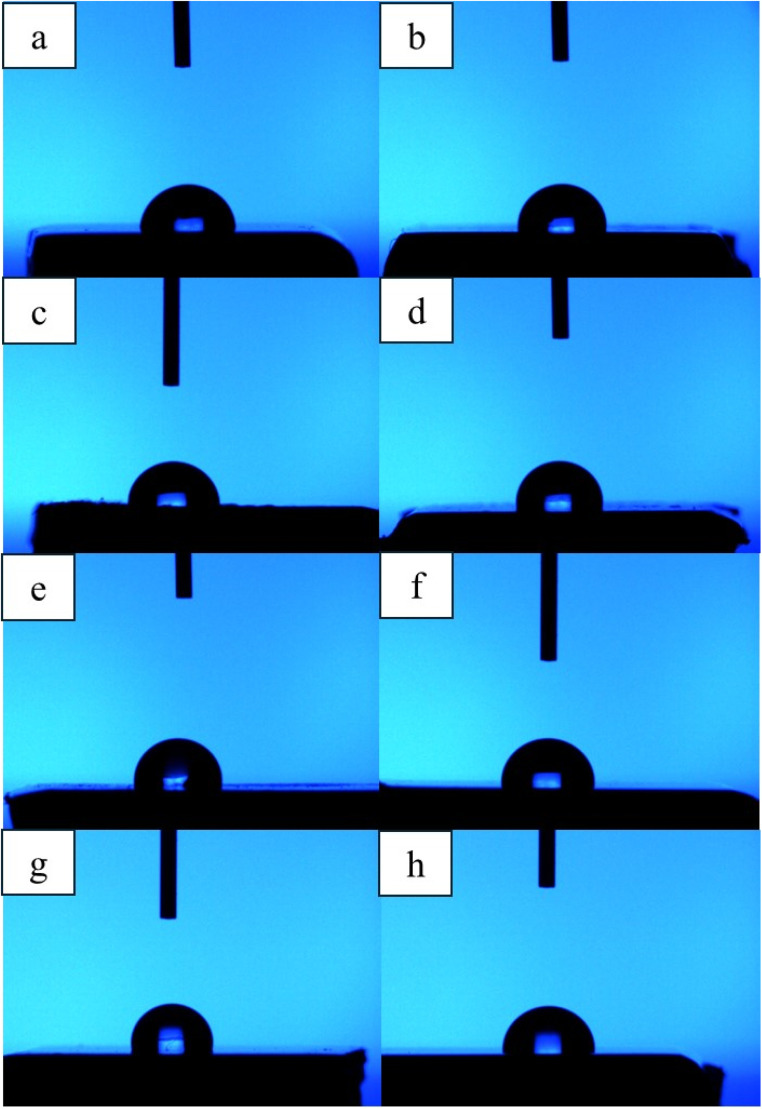
Contact angle measurement: (a) UHMWPE, (b–h) UHMWPE/graphene coating with E-beam at 0, 50, 100, 250, and 500 kGy, respectively.

The experiments in Table 4 and Fig. 5 found that the contact angle of water droplets on the surface (*θ*_water_) changed significantly when graphene was coated and different amounts of E-beam irradiation were applied to the UHMWPE surface. The UHMWPE uncoating (0 kGy) material has a water contact angle of 85.37°, which is in the range of moderately hydrophobic materials. When UHMWPE was coated with GNPs without irradiation, the contact angle was found to increase to 94.59°, indicating that GNPs play an essential role in enhancing the hydrophobicity of the surface. Because the structure of graphene has a repulsive force against water (low surface energy), water cannot spread well on the surface.^32^ The application of E-beam irradiation at different doses influenced the contact angle. Notably, at 50 kGy, the contact angle decreased slightly to 89.61°, which might be due to the graphene structure not being fully adhered to the surface, which is related to the coefficient of friction at 50 kGy. At 100 kGy, the point at which the material has the best overall properties in many aspects, the contact angle returns to 94.23°, indicating good adhesion of graphene and uniform distribution. With increasing irradiation doses of 150, 200, 250, and 500 kGy, the contact angle gradually increased until reaching a maximum of 97.50° at 500 kGy, indicating that the surface becomes increasingly hydrophobic. However, other properties such as strength or wear resistance may start to decrease due to chain scission.

**Table 4:** Contact angle results for the UHMWPE, UHMWPE/graphene coating with E-beam irradiation at different doses

Samples (E-beam content kGy)	*θ*_water_ (°)
UHMWPE uncoating (0 kGy)	85.37 ± 2.53
UHMWPE/GNPs coating (0 kGy)	94.59 ± 1.57
UHMWPE/GNPs coating (50 kGy)	89.61 ± 1.10
UHMWPE/GNPs coating (100 kGy)	94.23 ± 2.34
UHMWPE/GNPs coating (150 kGy)	94.31 ± 2.72
UHMWPE/GNPs coating (200 kGy)	95.01 ± 2.13
UHMWPE/GNPs coating (250 kGy)	97.39 ± 4.36
UHMWPE/GNPs coating (500 kGy)	97.50 ± 1.42

### 3.7 Proposed reaction mechanism of UHMWPE/graphene coating with E-beam irradiation

**Fig. 6:**
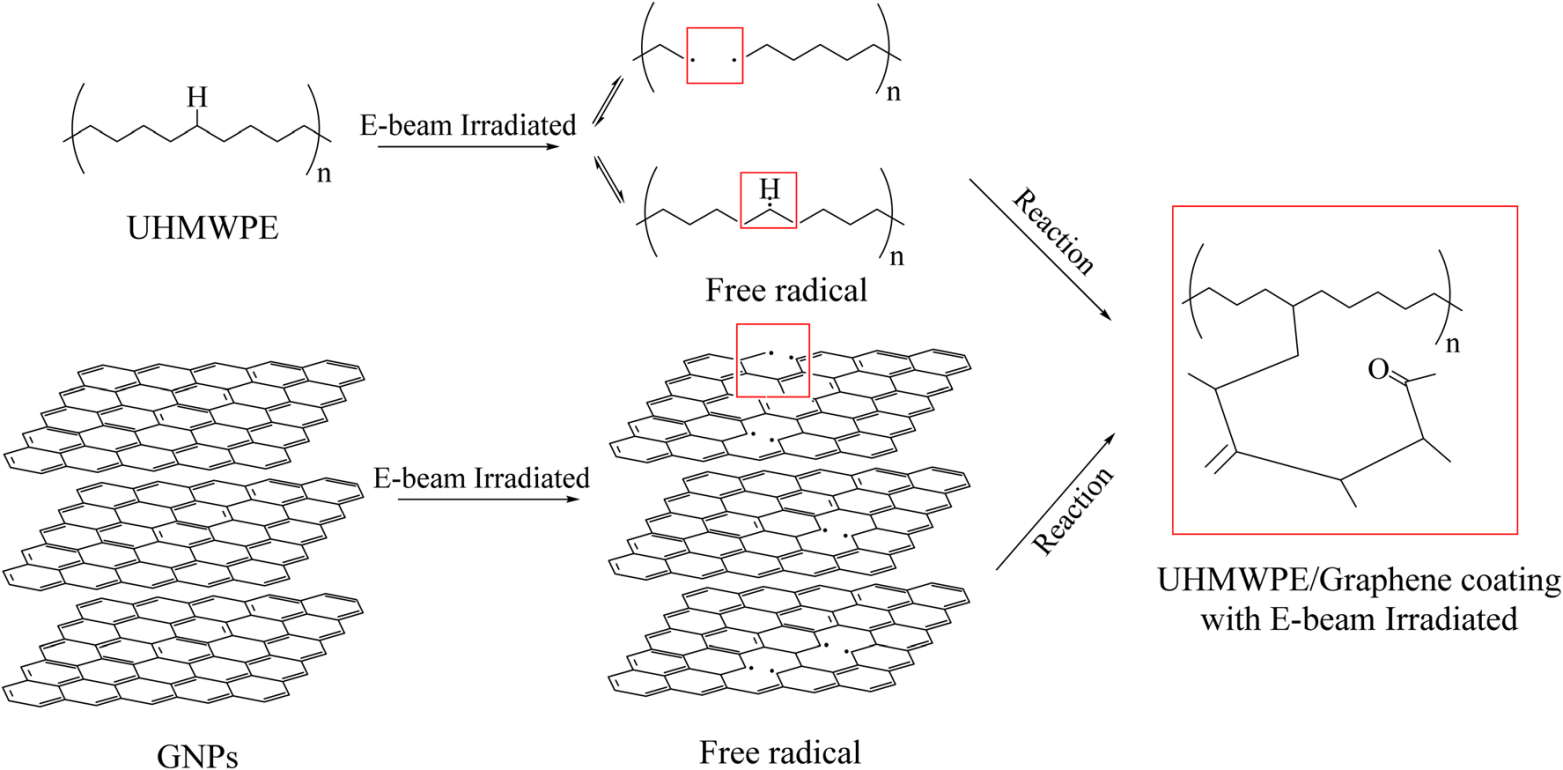
Proposed reaction mechanism of UHMWPE/graphene coating with E-beam irradiation.

The proposed reaction mechanism is presented in Fig. 6. When irradiated with E-beam, the C–C and C–H bonds of UHMWPE and GNPs are broken, generating free radicals that is able to form new bonds with each other or with reinforcing materials. The result is the formation of C–C bonds between the GNPs and the UHMWPE chains, which are chemical bonds that are significantly stronger than the original physical bonds.^33^ This results in significantly improved mechanical properties of the material, especially in terms of strength, wear resistance, and tensile strength. (C=O stretching vibration), which indicates the presence of oxygen-containing functional groups, such as ketones or carboxylic acids, in the material structure. It may occur after irradiation due to the oxidation process of UHMWPE itself. Although irradiation is carried out in vacuum, the material is still exposed to oxygen in air during sample preparation steps, such as during hot pressing or aging test in air.^22^

Table 5 presents a comparative analysis of UHMWPE composites reinforced with GNPs, highlighting the effects of different fabrication methods, filler contents, and property enhancements. The current study employed dip coating combined with E-beam irradiation using 1 wt% GNPs and achieved a hardness of 68 Shore D and a 38% reduction in the coefficient of friction (CoF) at 100 kGy. In comparison, Martínez-Morlanes *et al.*^4^ utilized 3–5 wt% GNPs through ball milling and hot pressing, reporting values of up to 60 Shore D in hardness and 25% CoF reduction. Shtertser *et al.*^34^ applied cyclic impact compaction with 0.5 wt% GNPs (NDC), resulting in limited changes in hardness (19.9 HBW) and a 10% CoF reduction, along with a noted decrease in tensile strength. Additionally, Xu *et al.*^31^ used electron beam irradiation up to 130 kGy on XLPE/GNPs composites with 15 wt% GNPs, leading to improvements in thermal conductivity and moderate increases in hardness and wear resistance, though surface resistivity was not reported. By contrast, the present study is the only one among those reviewed that investigated and achieved a significant reduction in surface resistivity from 5.33 × 10^15^ Ω to 2.15 × 10^8^ Ω at 100 kGy, demonstrating the effectiveness of combining surface engineering with low GNP loading, and offering performance characteristics that complement or exceed values previously reported in similar studies.^4,31^

**Table 5:** Comparison of the results of this research with other researches using UHMWPE materials combined with GNPs

Method	GNPs (% wt)	Hardness (Shore D)	Tensile strength (MPa)	Coefficient of friction (CoF)	Surface resistivity (Ω)	Ref.
Ball milling/blade mixer + hot pressing	0.1–5.0	60	No test	Decreased by approximately 25% at 3% wt GNPs	No test	4
PE/GNPs composites + extruder + E-beam 130 kGy	—	No test	27.06	No test	No test	31
UHMWPE/GNPs composites + cyclic impact compaction	0.5	63.6	22.00	Decreased by approximately 10% at 0.5% wt GNPs	No test	34
UHMWPE/GNPs dip coating + E-beam 100 kGy	1.0	68	28.94	Decreased by approximately 38% at 1% wt GNPs	Decreased from 5.33 × 10^15^ to 2.15 × 10^8^ at 100 kGy	This work

## 4 Conclusions

The study demonstrated that surface modification of Ultra-High Molecular Weight Polyethylene (UHMWPE) through the dip coating of 1 wt% GNPs and subsequent electron beam irradiation (E-beam) significantly enhanced the tribological, mechanical, electrical, and surface characteristics of the material. Among the tested doses, irradiation at 100 kGy provided the most optimal improvements, yielding the lowest coefficient of friction (0.1793), highest tensile strength (28.94 MPa), enhanced elongation at break (58.35%), and maximum hardness (68 Shore D). The electrical surface resistivity also dropped markedly to 2.15 × 10^8^ Ω, indicating improved surface conductivity. FTIR analysis revealed the formation of C=O bonds from oxidation after irradiation, and SEM images confirmed improved graphene adhesion at moderate doses. However, increasing the irradiation beyond 100 kGy led to property degradation due to excessive chain scission, which negatively impacted mechanical integrity and electrical conductivity. Therefore, the synergistic effect of GNPs and controlled E-beam exposure, particularly at 100 kGy, offers a promising strategy for tailoring UHMWPE for advanced applications in biomedical devices, electronics, and wear-resistant components.

## Conflicts of interest

There are no conflicts to declare.

## Data Availability

The datasets generated during and/or analysed during the current study are available from the corresponding author on reasonable request.
